# Supplementary material for: Synthesis and Modeling Studies of Furoxan Coupled Spiro-Isoquinolino Piperidine Derivatives as NO Releasing PDE 5 Inhibitors
Source: Biomedicines. 2020 May 14;8(5):121. doi: 10.3390/biomedicines8050121 (PMC7277557; doi:10.3390/biomedicines8050121)

Supplementary File 1

^{1}HNMR, 13C NMR and HMRMS, mass spectra of compound at different steps of the synthesis.

Mass spectra of **3**

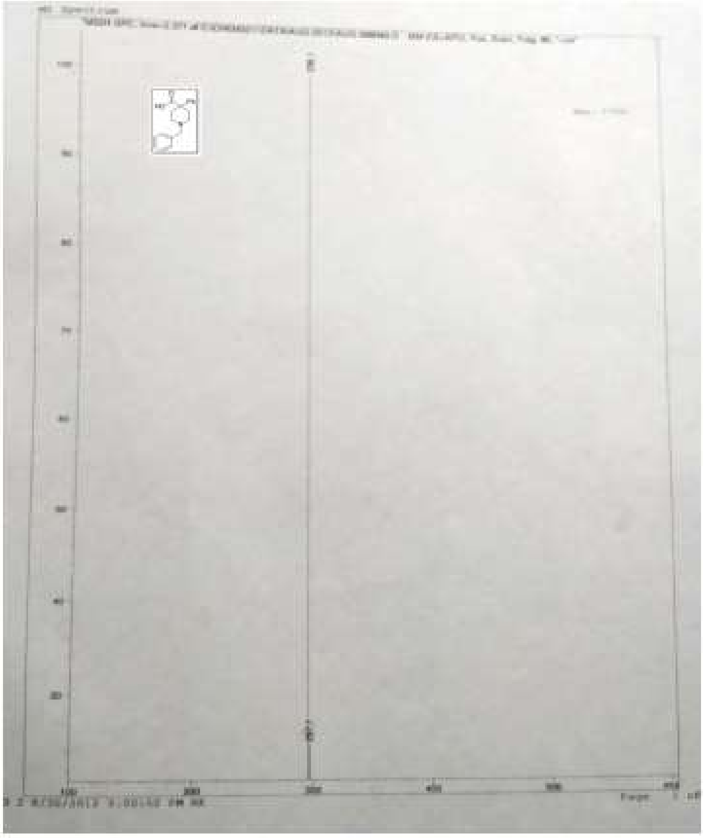

Mass spectra of **6**

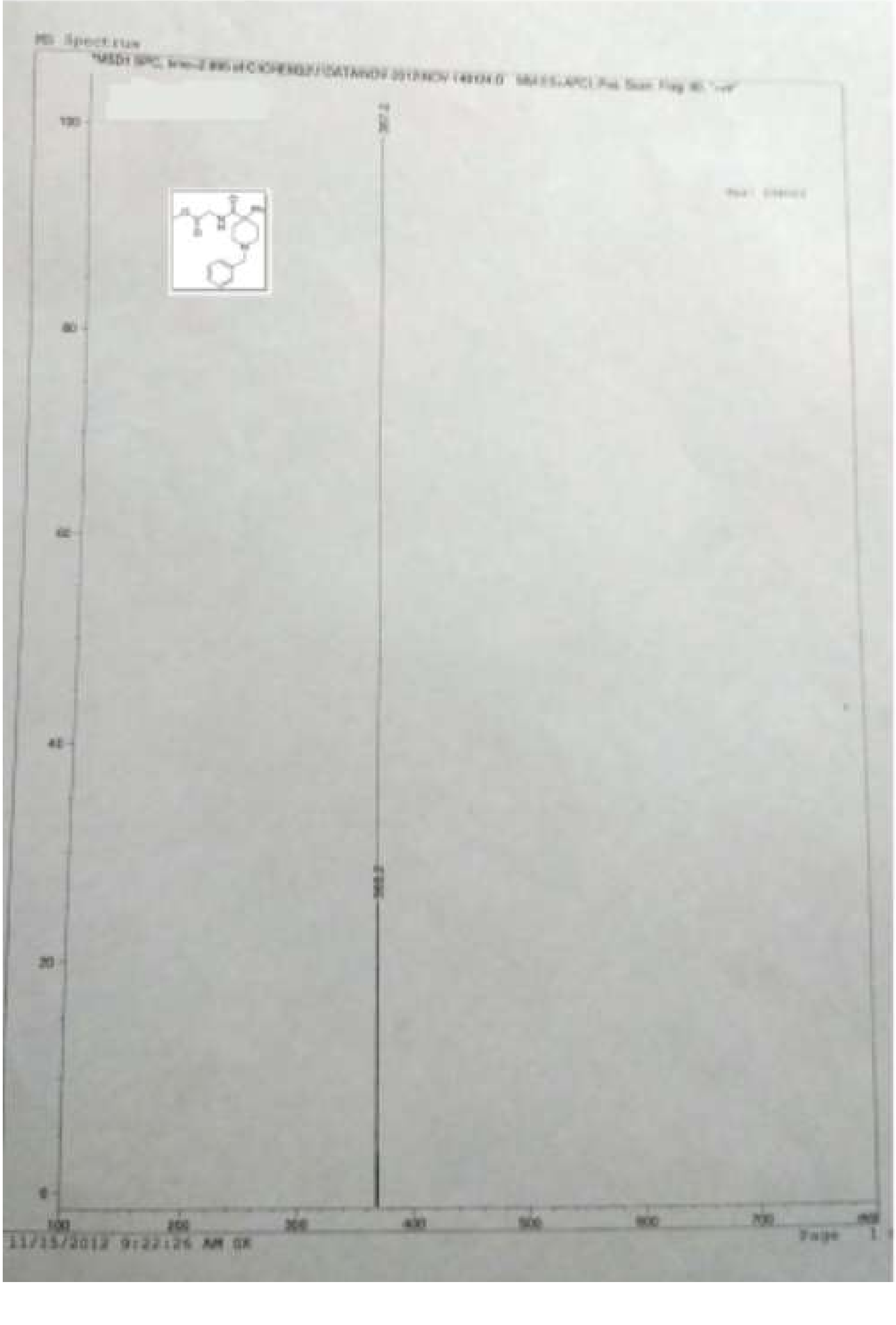

Mass spectra of **7**

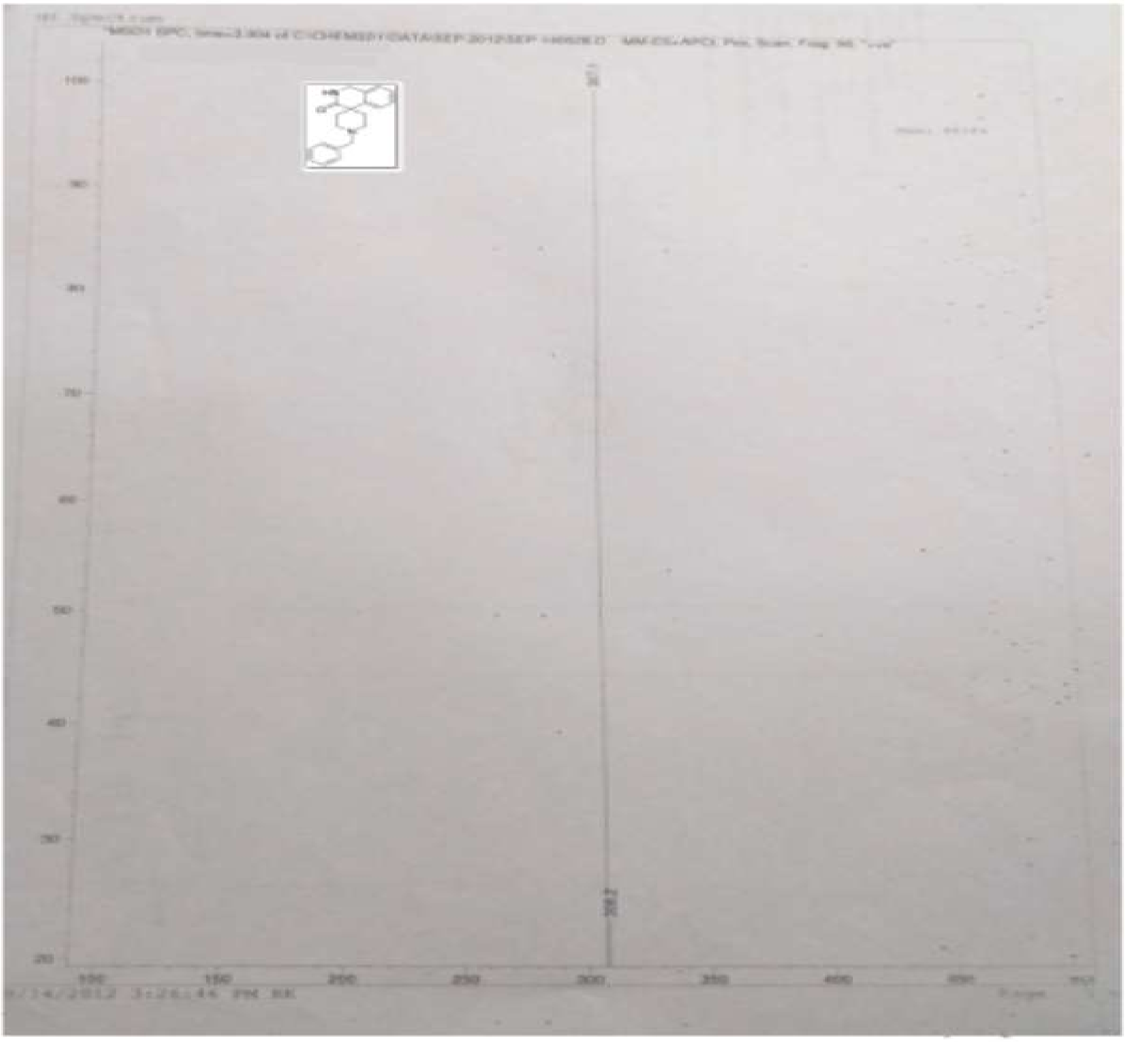

1HNMR spectra of **7**

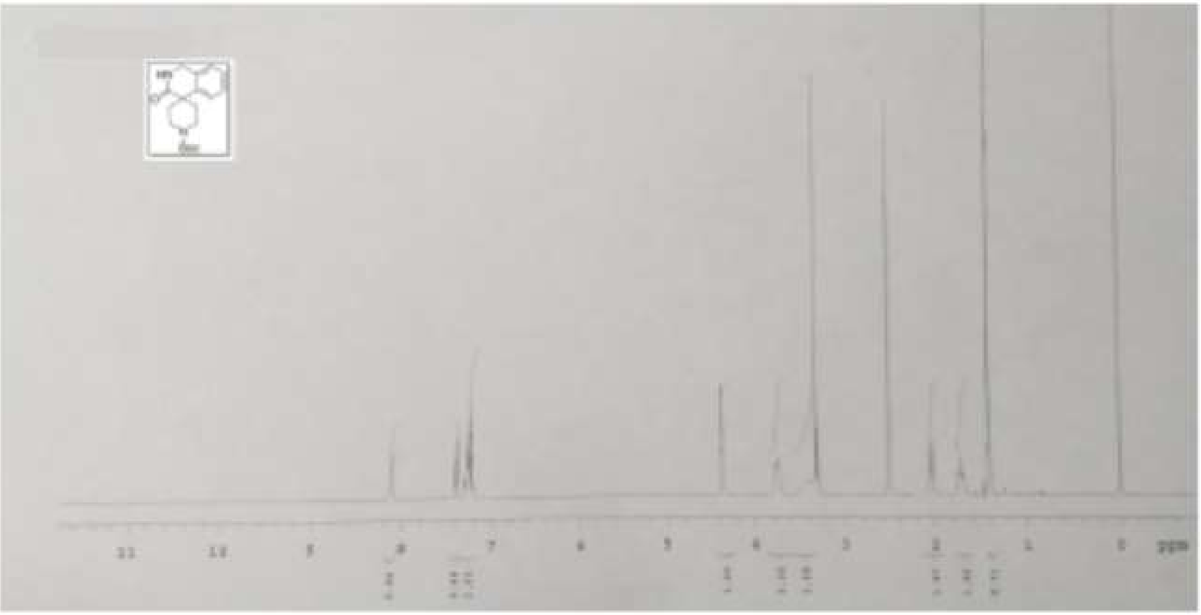

1HNMR spectra of **10a**

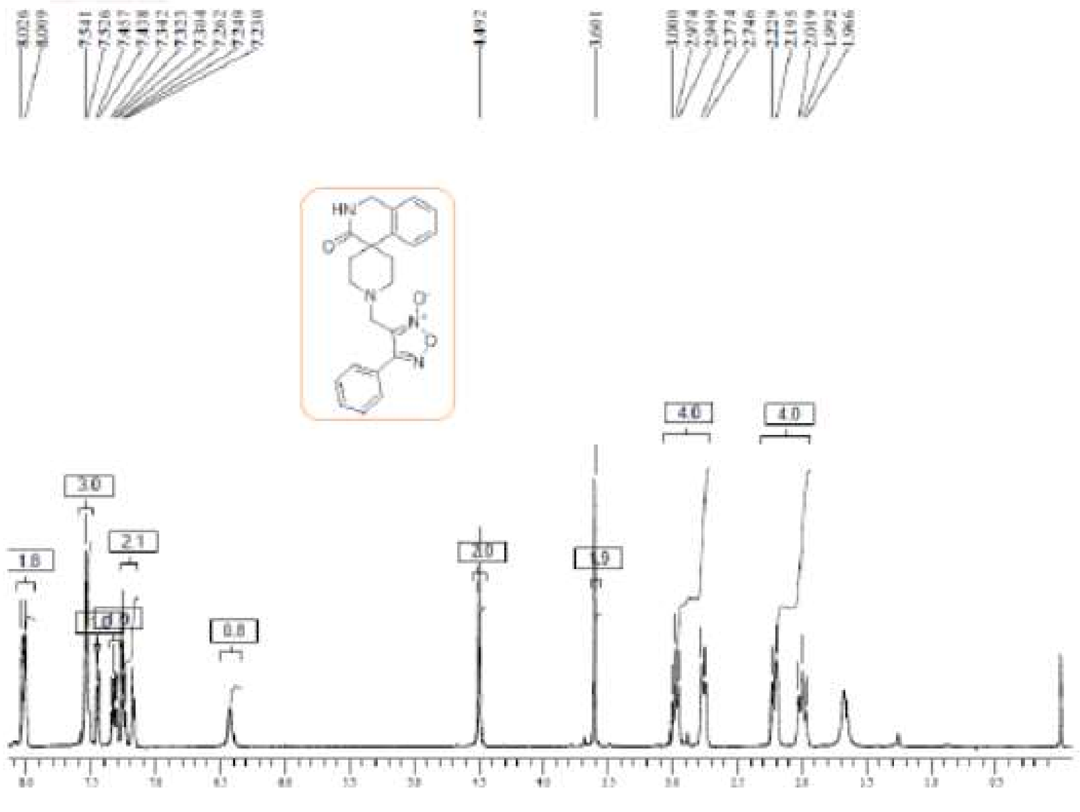

13C NMR spectra of **10a**

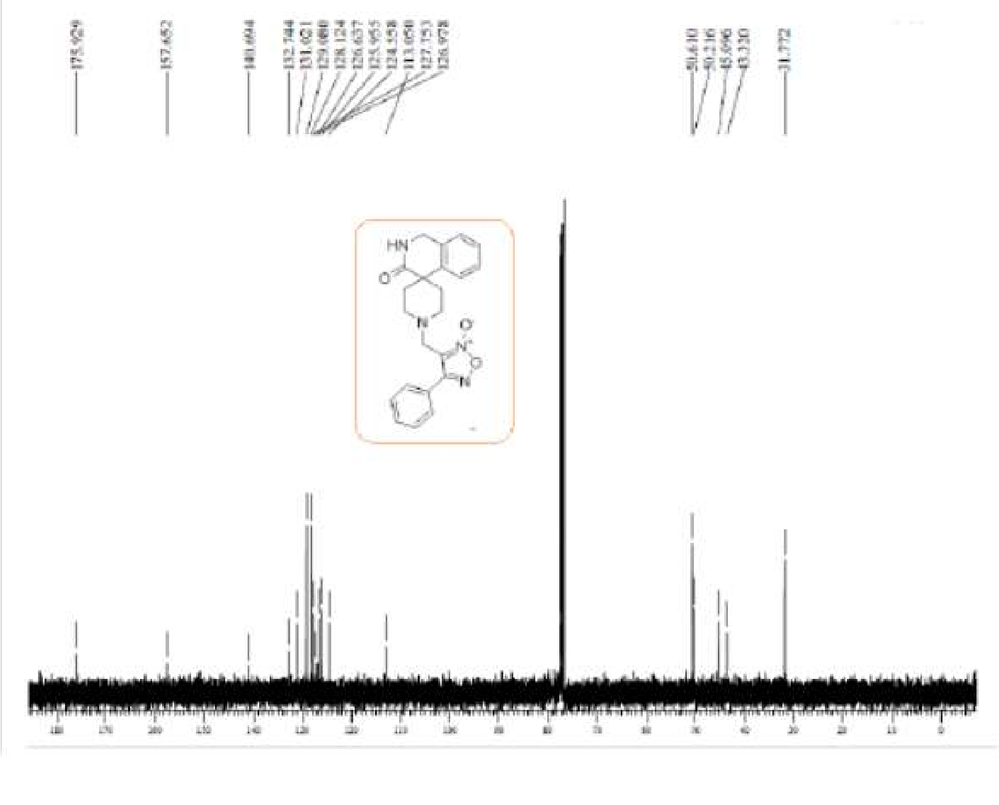

Mass spectra of **10a**

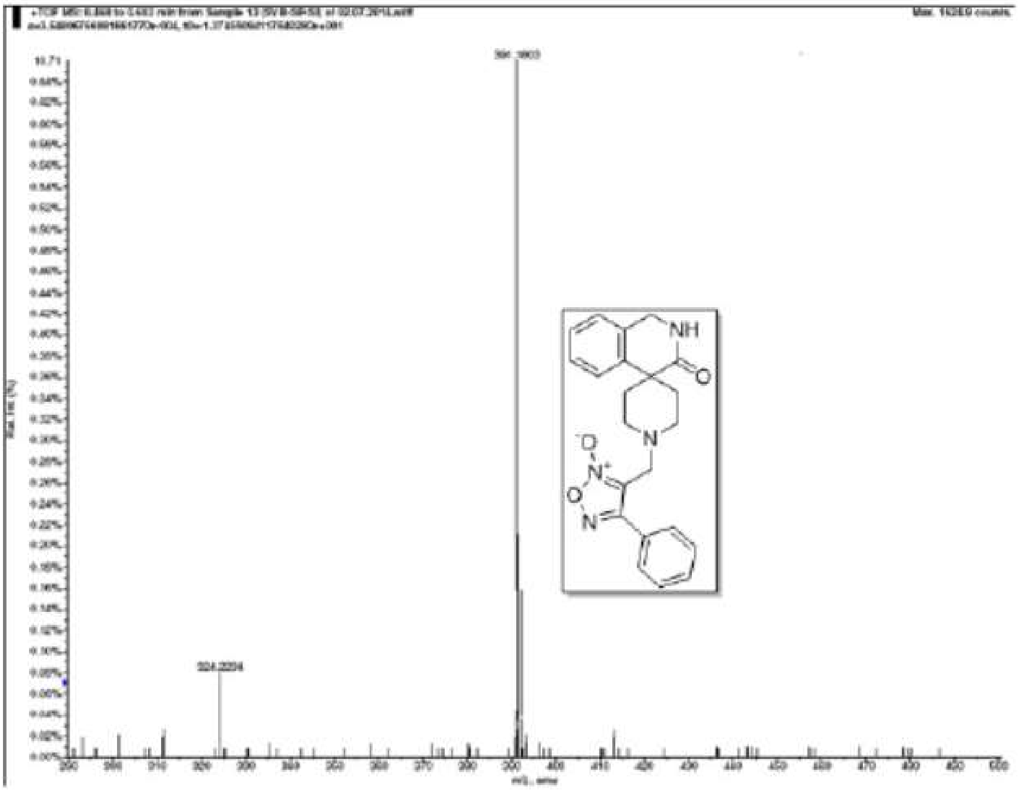

1HNMR spectra of **10b**

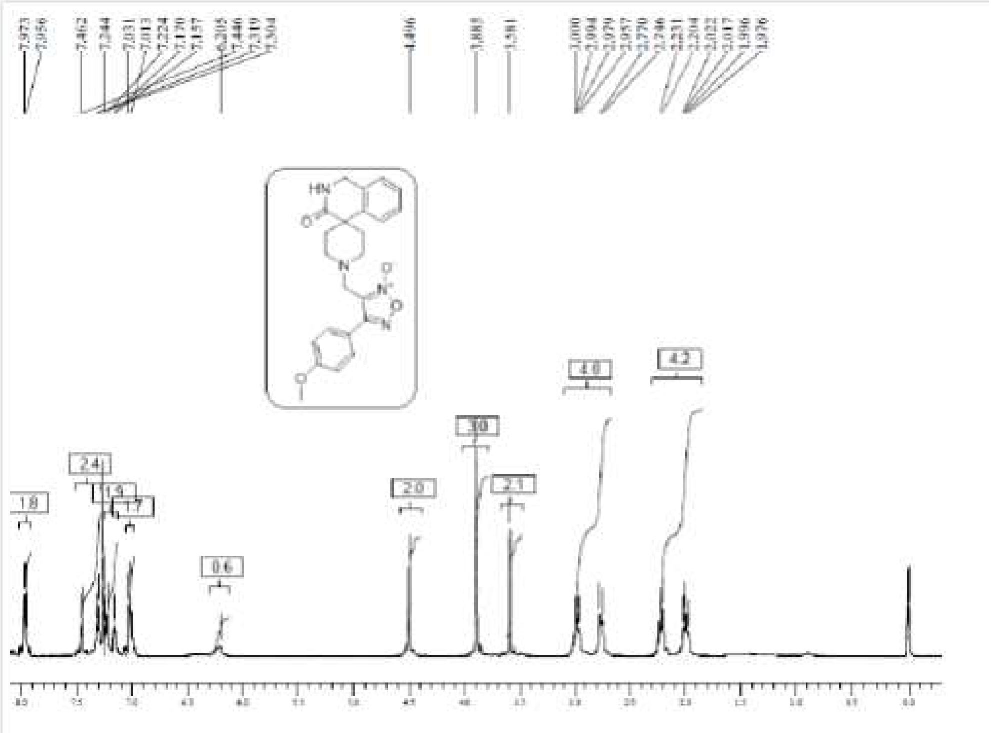

13C NMR spectra of **10b**

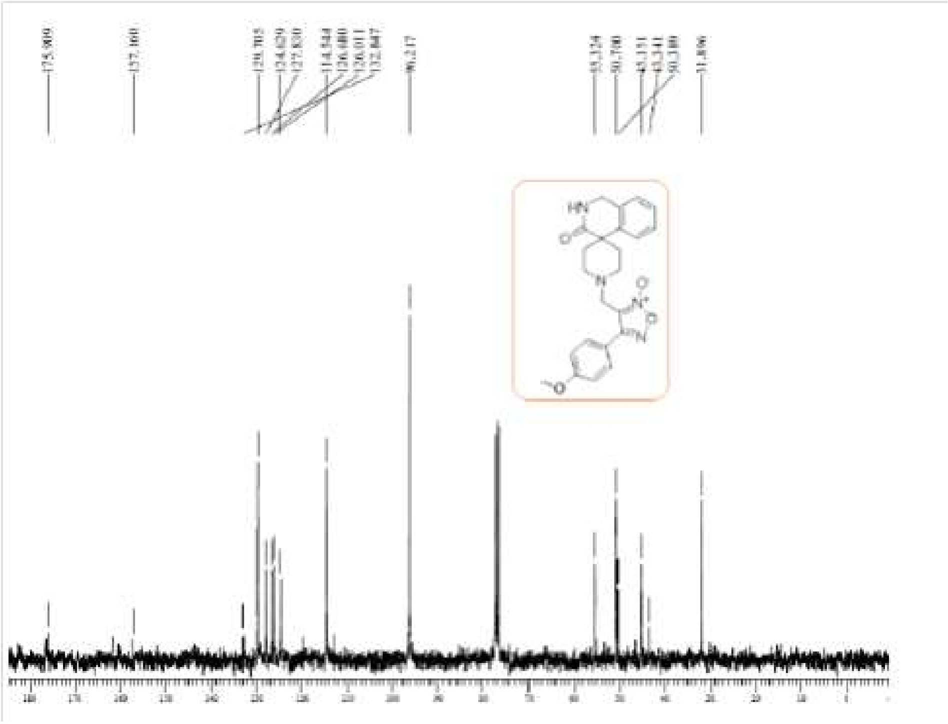

HRMS spectra of **10b**

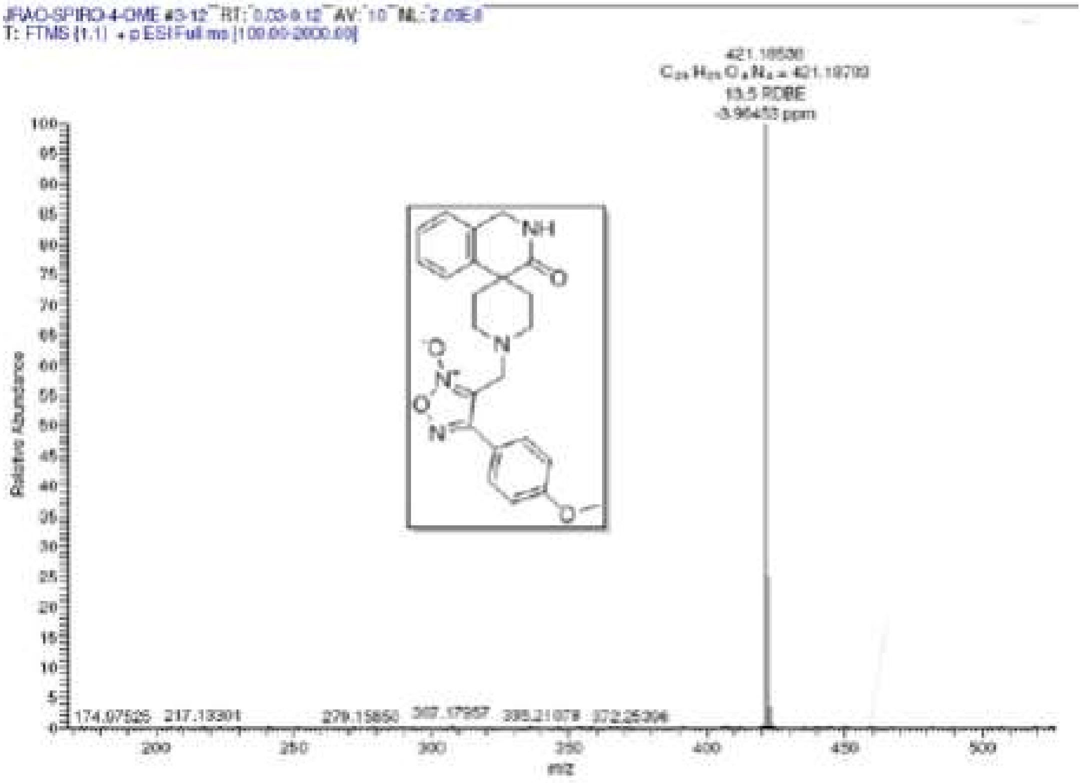

1HNMR spectra of **10c**

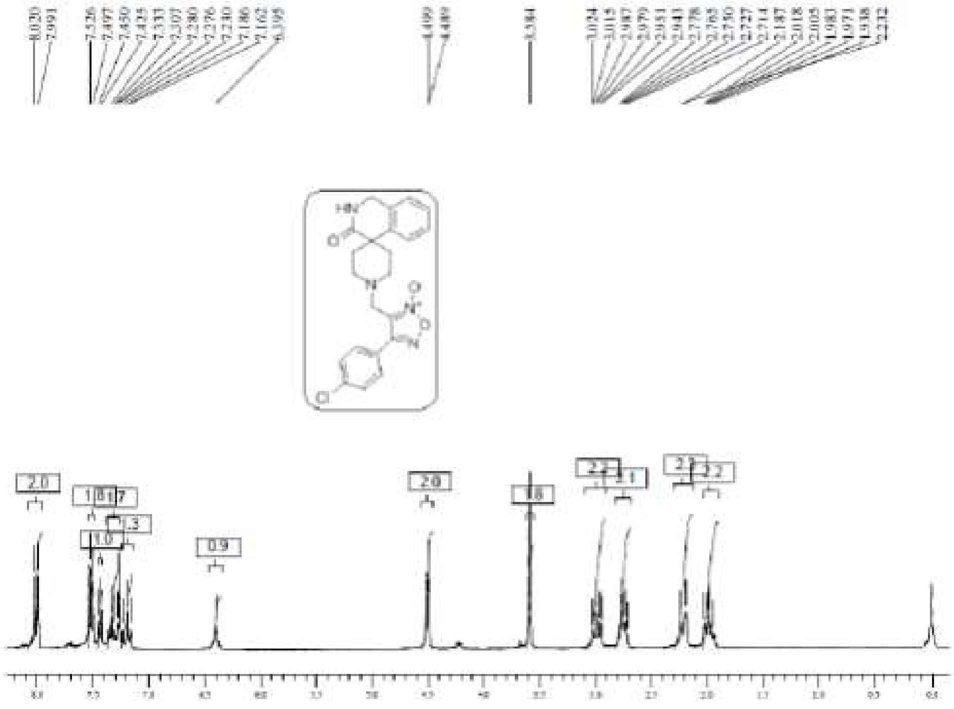

13C NMR spectra of **10c**

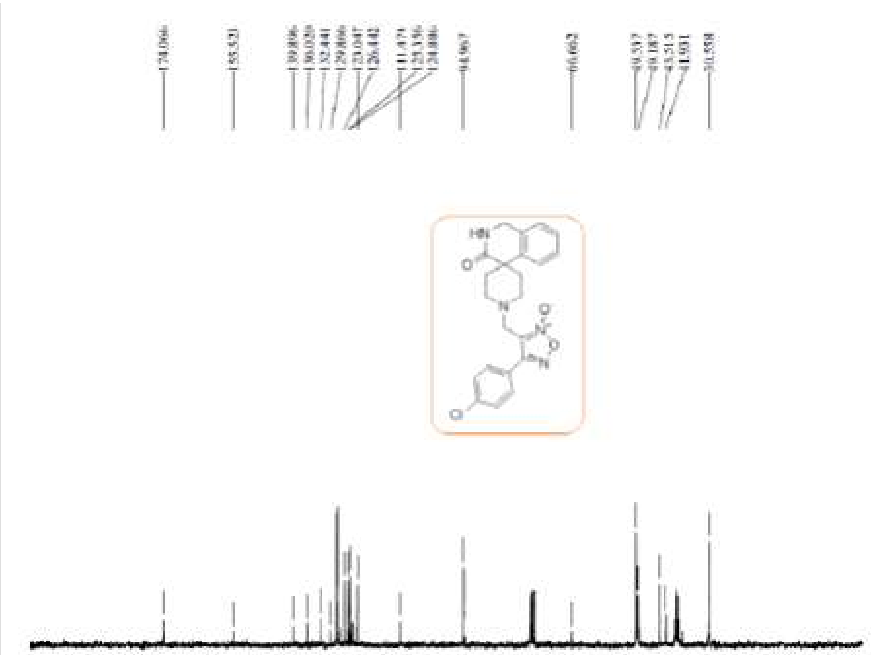

1HNMR spectra of **10d**

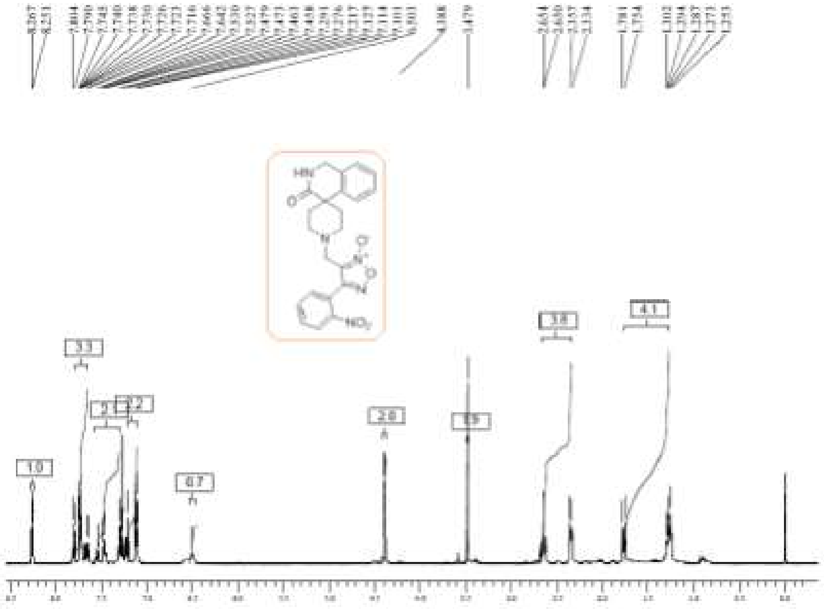

13C NMR spectra of **10d**

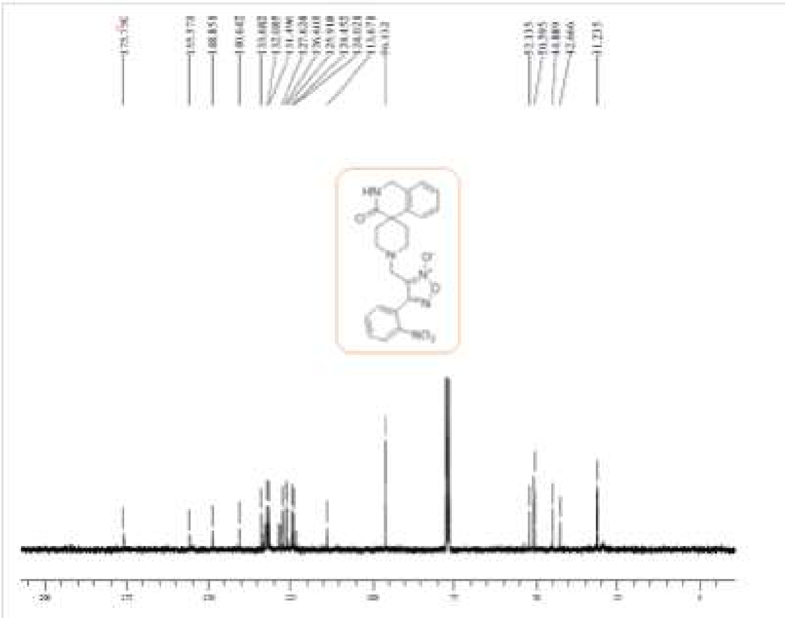

HRMS spectra of **10d**

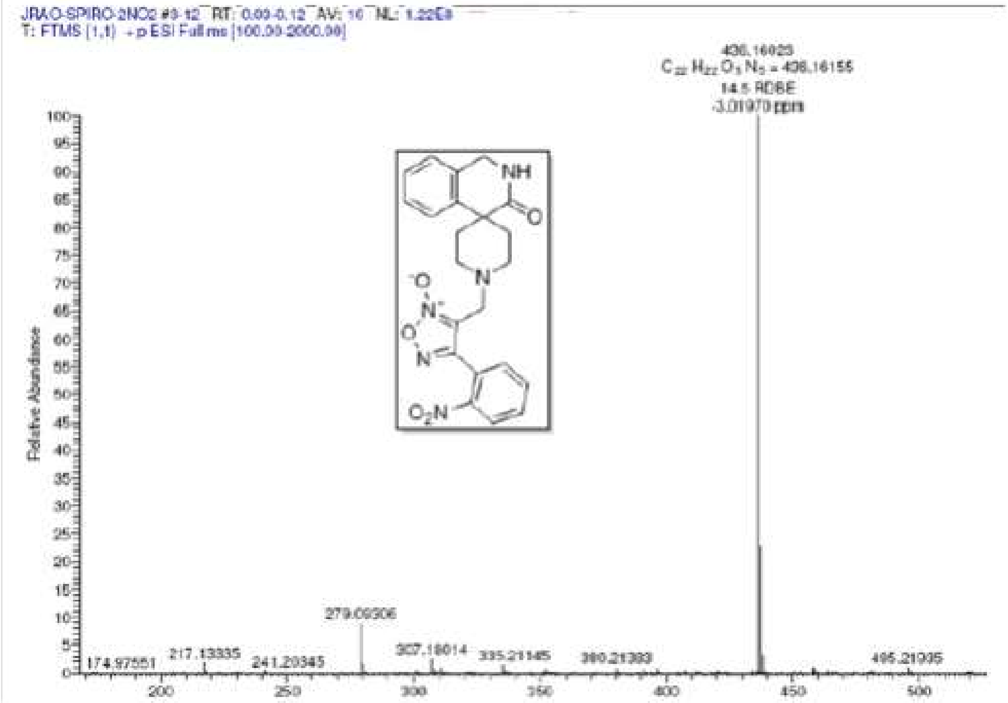

HRMS spectra of **10e**

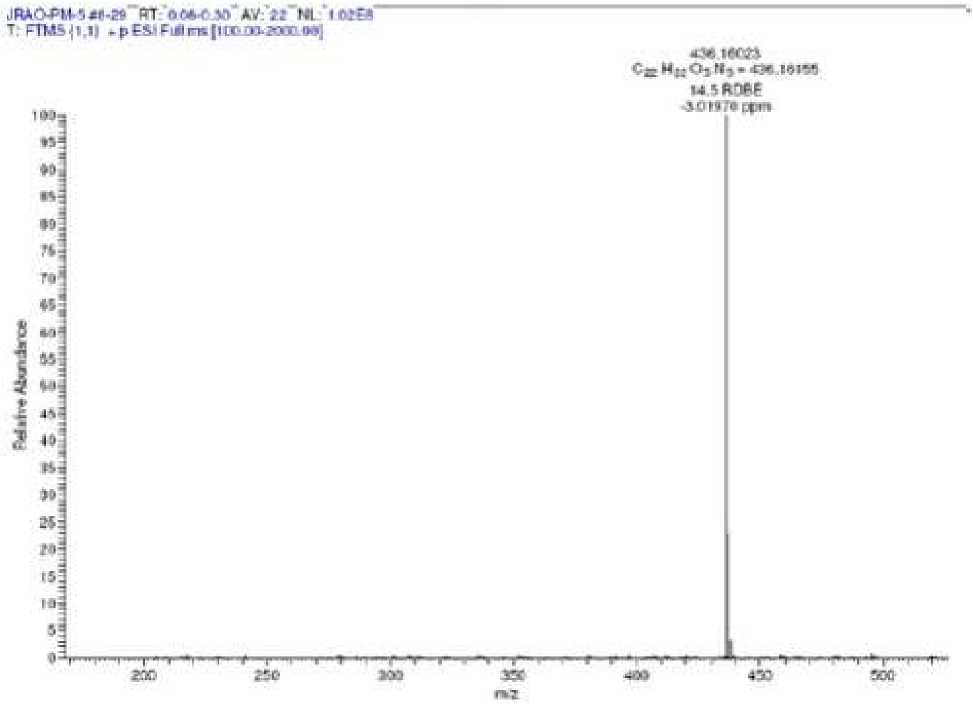

1H NMR spectra of **10f**

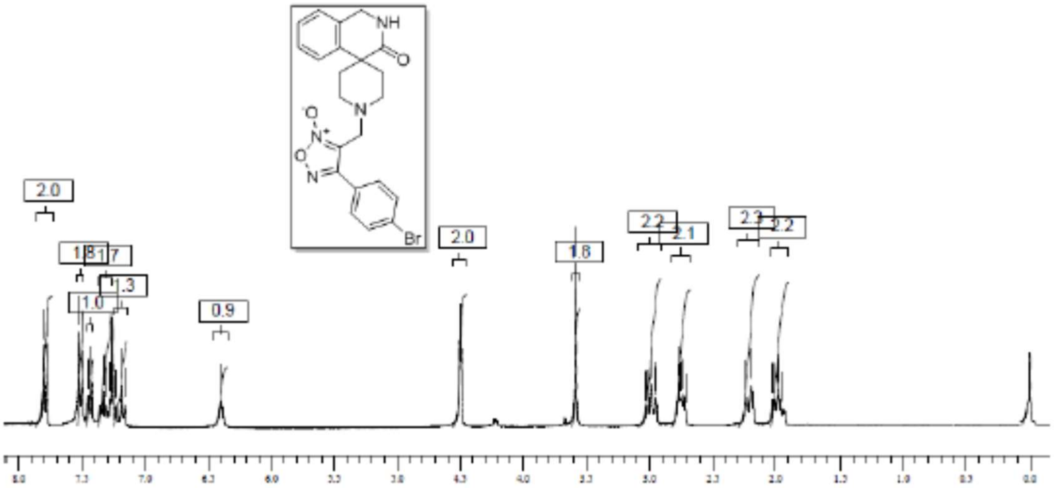

13C NMR spectra of **10f**

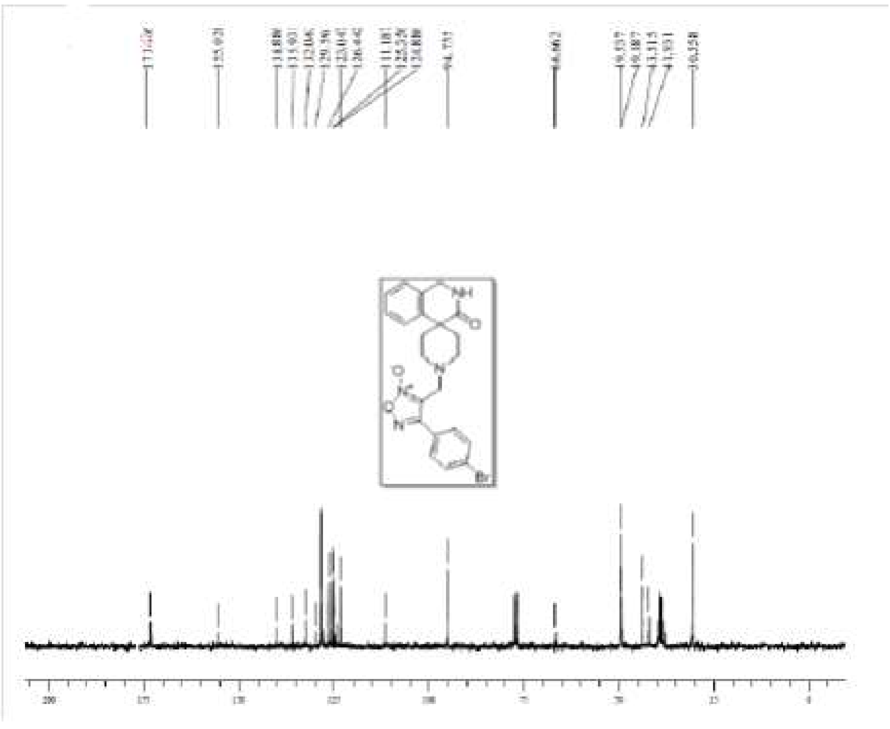

HRMS spectra of **10g**

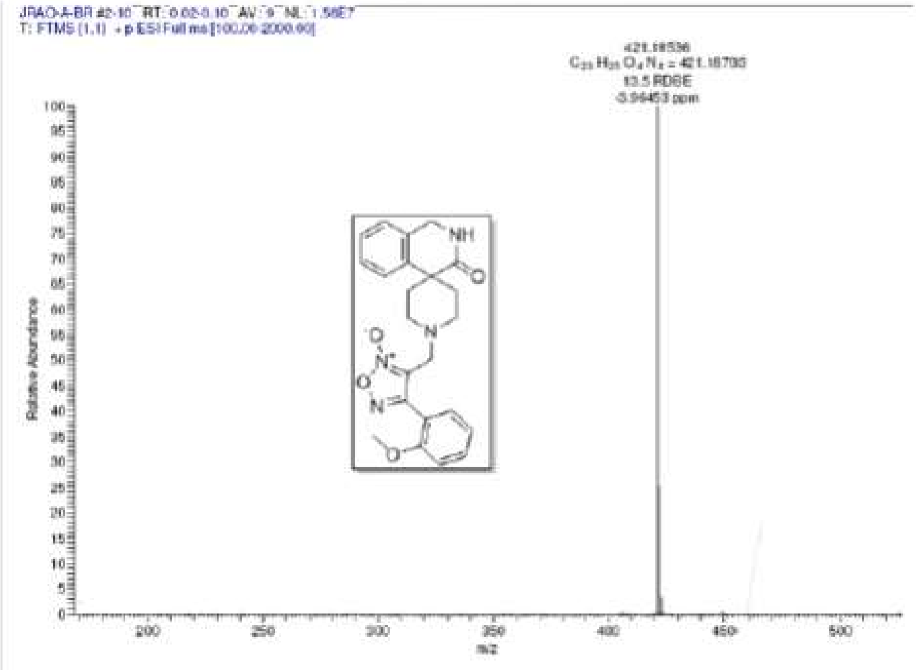

HRMS spectra of **10h**

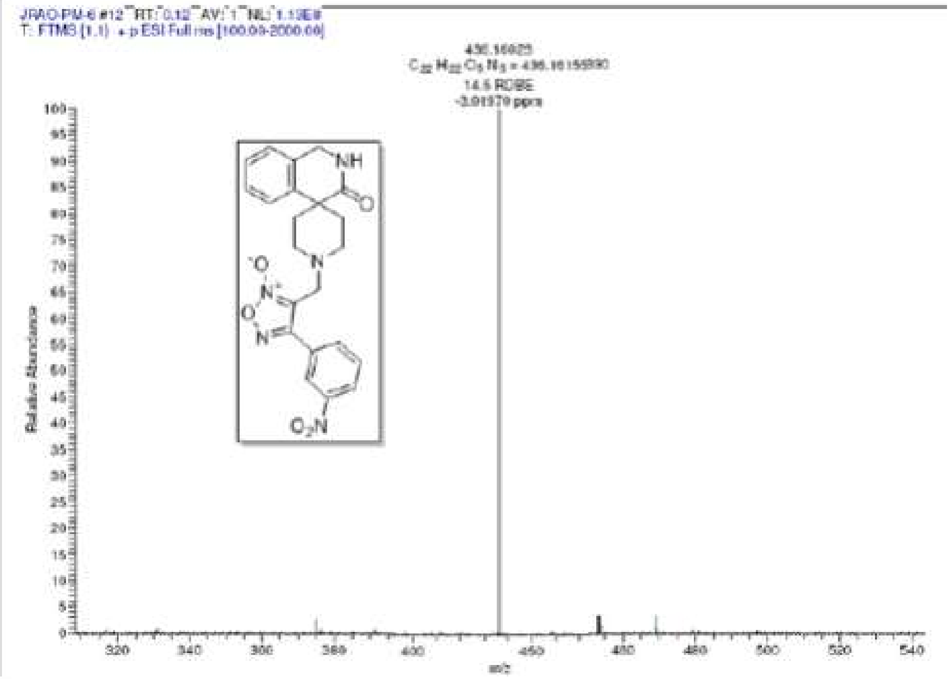

HRMS spectra of **10i**

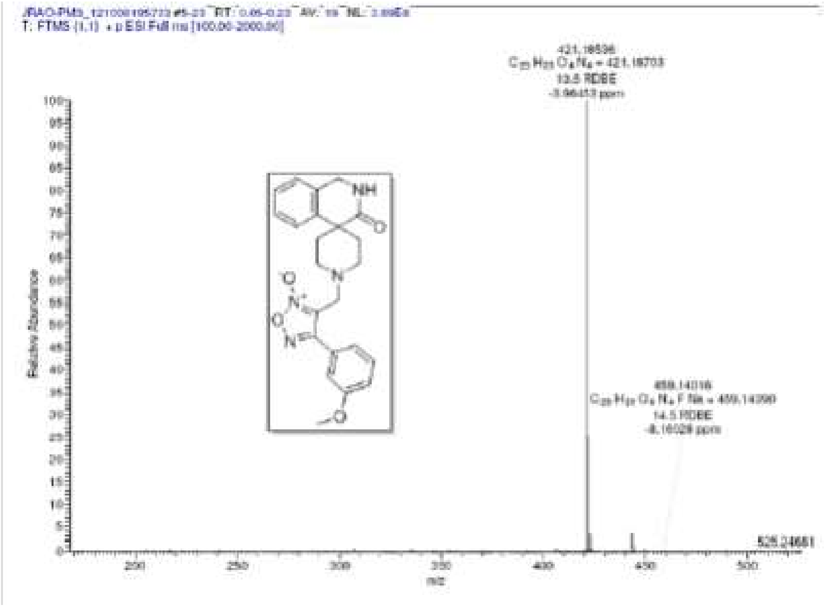

Supplement: Supplementary file 1 [file biomedicines-08-00121-s001.pdf]
